# SHP2 regulates adipose maintenance and adipocyte-pancreatic cancer cell crosstalk via PDHA1

**DOI:** 10.1007/s12079-022-00691-1

**Published:** 2022-09-08

**Authors:** Appolinaire A. Olou, Joe Ambrose, Jarrid L. Jack, McKinnon Walsh, Mariana T. Ruckert, Austin E. Eades, Bailey A. Bye, Prasad Dandawate, Michael N. VanSaun

**Affiliations:** Department of Cancer Biology, University of Kansas Medical Center, 3901 Rainbow Boulevard, Kansas City, KS 66160 USA

**Keywords:** SHP2, PDHA1, ROS, Adipocytes, Cancer

## Abstract

Adipocytes are the most abundant cell type in the adipose tissue, and their dysfunction is a significant driver of obesity-related pathologies, such as cancer. The mechanisms that (1) drive the maintenance and secretory activity of adipocytes and (2) mediate the cancer cellular response to the adipocyte-derived factors are not fully understood. To address that gap of knowledge, we investigated how alterations in Src homology region 2-containing protein (SHP2) activity affect adipocyte function and tumor crosstalk. We found that phospho-SHP2 levels are elevated in adipose tissue of obese mice, obese patients, and differentiating adipocytes. Immunofluorescence and immunoprecipitation analyses as well as in-silico protein–protein interaction modeling demonstrated that SHP2 associates with PDHA1, and that a positive association promotes a reactive oxygen species (ROS)-driven adipogenic program. Accordingly, this SHP2-PDHA1-ROS regulatory axis was crucial for adipocyte maintenance and secretion of interleukin-6 (IL-6), a key cancer-promoting cytokine. Mature adipocytes treated with an inhibitor for SHP2, PDHA1, or ROS exhibited an increased level of pro-lipolytic and thermogenic proteins, corresponding to an increased glycerol release, but a suppression of secreted IL-6. A functional analysis of adipocyte-cancer cell crosstalk demonstrated a decreased migration, invasion, and a slight suppression of cell cycling, corresponding to a reduced growth of pancreatic cancer cells exposed to conditioned media (CM) from mature adipocytes previously treated with inhibitors for SHP2/PDHA1/ROS. Importantly, PDAC cell growth stimulation in response to adipocyte CM correlated with PDHA1 induction but was suppressed by a PDHA1 inhibitor. The data point to a novel role for (1) SHP2-PDHA1-ROS in adipocyte maintenance and secretory activity and (2) PDHA1 as a regulator of the pancreatic cancer cells response to adipocyte-derived factors.

**Supplementary Information:**

The online version contains supplementary material available at 10.1007/s12079-022-00691-1.

## Introduction

The adipose tissue is a major source of cancer-promoting signals (Zyromski et al. 2009; Rebours et al. 2015). The link between the adipose tissue and cancer involves immune cells, such as macrophages (Mayi et al. 2012; Wunderlich et al. 2018; Tiwari et al. 2019), lymphocytes (Del Corno et al. 2018; Wunderlich et al. 2018), neutrophils (Quail et al. 2017; Gummlich 2021), and may involve non-immune cells as well. As the most abundant non-immune cell component of the adipose tissue, studies demonstrated that adipose tissue cells, or adipocytes, are sufficient to drive the progression of multiple cancer types (Nieman et al. 2011; Huang et al. 2017; Wu et al. 2019). Targeting adipocyte-cancer crosstalk requires an understanding of adipocyte maintenance and the identification of adipocyte-derived molecular signals that drive functional cancer cell responses.

Mitochondria are autonomous organelles that facilitate cellular energetics and energy metabolism. Disruption of normal mitochondrial function is associated with pathologies such as the development/progression of obesity, inflammation, and/or cancer (Ahn and Metallo 2015). One key function of mitochondria is pyruvate metabolism to acetyl coenzyme A, mediated by Pyruvate dehydrogenase alpha subunit 1 (PDHA1) (Zhou et al. 2001; Sun et al. 2015; Cai et al. 2020). Acetyl coenzyme A serves as a substrate for a cascade of reactions leading to ATP generation or lipogenesis (Sun et al. 2015; Cai et al. 2020). This process also is a major source of reactive oxygen species (ROS) generation (Li et al. 2013). PDHA1 inhibition and subsequent disruption of pyruvate metabolism and ROS generation was correlated with a reduced inflammatory response in mice (Li et al. 2017). Importantly, elevated levels of ROS have been found in obese adipose tissue as well as during adipogenesis (Furukawa et al. 2004). However, mediators of ROS-driven adipocyte maintenance as well as the function of this mechanism in adipocyte-cancer cells crosstalk are not fully known.

SHP2 is a tyrosine phosphatase involved in a multitude of cellular processes such as development, differentiation (Feng 2007; Wang et al. 2021), and metabolism (Zhang et al. 2004; Niogret et al. 2019). Adipose specific deletion of SHP2 was reported to result in loss of fat (He et al. 2013). Alternatively, SHP2 is also a proto-oncoprotein known for its intrinsic role in cancer initiation and progression (Ruess et al. 2018). In fact, pancreas-specific deletion of SHP2 was reported to inhibit pancreatic intraepithelial neoplasia (PanIN) formation and prevent pancreatic cancer development (Ruess et al. 2018). While the targeting of SHP2 for tumor intrinsic signaling is ongoing, the effect of SHP2 inhibition in adipocytes has not been investigated with respect to adipocyte-tumor crosstalk. Yet, a fatty infiltration (steatosis) of intra as well inter-pancreatic adipose cells increased adipocyte-pancreatic cancer crosstalk (Mendonsa et al. 2015) and correlated with metastatic disease (Mathur et al. 2009). While adipocyte-derived factors such as adipokines can promote pancreatic cancer progression (Mendonsa et al. 2015), the processes that regulate the secretion of those adipokines as well as the mechanisms by which the adipokines, once secreted, drive pancreatic cancer are not completely understood. The current study implicated a SHP2-PDHA1-ROS regulatory axis in adipocyte maintenance and adipocyte-tumor cells crosstalk.

## Materials and methods

### Cell lines, culture conditions, and reagents

3T3-L1 pre-adipocytes (cat# CL-173) and adipose-derived mesenchymal stem cells (adMSC, cat# PCS-500-011) were obtained from ATCC. The murine pre-adipocytes were maintained in high glucose (4.5 g/L) Dulbecco’s Modified Eagle Media, DMEM, (ThermoFisher Scientific) supplemented with 10% heat-inactivated bovine calf serum (R&D Systems-Atlanta Biologicals) and antibiotic–antimycotic (ThermoFisher Scientific) whereas the human pre-adipocytes were maintained in mesenchymal stem cell growth media (cat# PCS-500-030 and PCS-500-040, ATCC). K8484 and Panc10.05 cell lines have been previously described (Manley et al. 2022). Acetyl Coenzyme A, Nitro Blue Tetrazolium (NBT), N-Acetyl Cysteine (NAC) and H_2_O_2_ were purchased from Sigma-Aldrich. 2X SYBR green master mix (cat# K1070) was purchased from APExBIO (Houston, TX). SHP099 (cat. # S8278) and CPI-613 (cat# S2776; PDHA1 inhibitor) were from Selleckchem. For drug/compound treatment, a stock solution of 40 mM of SHP099 or 200 mM PDHA1 inhibitor was prepared in DMSO and further diluted in water (1:1 ratio) before the desired final concentrations were reconstituted in the media; appropriate volume of 50% DMSO (in water) was used as vehicle control throughout the experiments. NAC was initially dissolved in water. Where the same vehicle treatment control was used for NAC, SHP099 and PDHA1 inhibitor, NAC stock solution was diluted in DMSO (1:1 ratio) before the final desired concentrations were reconstituted in the culture media. The treatment media was replenished every 36–48 h.

### Adipogenesis stimulation

For induction of adipogenesis, 2 days over-confluent pre-adipocytes were switched from their normal growth media to differentiation media consisting of high glucose DMEM supplemented with 10% FBS, Insulin (cat# I0516, Sigma; 1 µg/ml for 3T3-L1, 3 µg/ml for adMSC), Dexamethasone (cat# 11015 Cayman Chemicals; 10 µg/ml), Rosiglitazone (cat# 71740, Cayman Chemicals; 3 µg/ml) and IBMX (cat# I5879, Sigma; 10 µg/ml) for 2 days (4 days for adMSC) before being maintained in DMEM with 10% FBS containing Insulin only (1 µg/ml); the media was changed every two days thereafter.

### Collection of conditioned media (CM)

At indicated time points, adipocyte culture media was replaced with serum-free media or, where treatments took place, the cells were first washed with 1X PBS, and cultured for 24 h. This media was then supplemented with fresh serum-free media at 1:1 ratio for downstream applications.

### Immunoblotting

Immunoblotting has been previously described (Messaggio et al. 2017). Protein samples were quantified using BCA (Pierce). 20–30 µg of protein were loaded per lane. Antibodies against pSHP2 [Y542 (cat# 3751)], SHP2 (cat# 3397), PDHA1 (cat# 3205), HK1 (cat# 2024), Lamin B1 (cat# 12586), p-AMPKα [Thr 172 (cat# 2535)], UCP1 (cat#14670), Perilipin-1 (cat# 9349), beta-actin (cat# 4970) and GAPDH (cat# 5174) were purchased from cell signaling technologies. Glut1 was from Invitrogen (cat# PA1-46152). Tubulin (cat# E7-s) was purchased from the Developmental Studies Hybridoma Bank (DSHB, Iowa City, IA).

### Oil red O staining

For Oil Red O staining, the cells were rinsed with 1X PBS, fixed in formalin for 45 min, rinsed again with 60% Isopropanol before being stained with Oil Red O (cat# A12989, Alfa Aesar; 5 mg/ml) in 60% Isopropanol for 15 min. The stained cells were washed in 60% Isopropanol at least 3 times, 15 s each, and left in distilled water for visualization under the microscope.

### Mitochondrial isolation and immunoprecipitation (IP) of SHP2

The protocol used to isolate the mitochondria has been previously established and reported (Cai et al. 2020). In brief, cells were lysed in MTiso-buffer [3 mM HEPES (pH 7.4), 210 mM mannitol, 70 mM sucrose and 0.2 mM EGTA] by Dounce homogenization [about 50 strokes (up and down)]. The homogenate was piled up on 340 mM sucrose solution in a 50 ml-tube, and centrifuged at 500×*g* (10 min, 4 °C) to remove nuclei and unbroken cells as pellet. Then, the supernatant was collected in 1.5 ml-tubes and centrifuged at 10,000×*g* (10 min, 4 °C) to isolate mitochondria as pellet. IP has been previously described (Olou et al.^2017^) with a modification. Briefly, the isolated mitochondrial pellet was lysed in CHAPS buffer {0.3% of 3-(3-cholamidopropyl)-dimethylammonio)-1-propanesulfonate, (CHAPS), 20 mM Tris–HCL (pH7.4), 120 mM NaCl, 10% glycerol and 5 mM EDTA) and the lysate was incubated at 4 degrees overnight with SHP2 or IgG antibodies crosslinked to the Dynabeads (Thermofisher).

### In-silico protein–protein interaction modeling

The protein structures of SHP2 (PDB ID: 2SHP) and PDHA1 (PDB ID: 1NI4) were downloaded from the protein data bank. These PDB files were prepared for docking by removing ligands, water molecules and additional chain of amino acids. Chain A was selected for both proteins. We further used GrammX protein–protein docking webserver v.1.2.0 (http://vakser.compbio.ku.edu/resources/gramm/grammx/) (Tovchigrechko and Vakser 2005, 2006) to predict the binding between SHP2 and PDHA1 by using default settings. About 10 models of the protein–protein complex were studied. The best predicted model was selected based on the number of hydrogen bonds, bond distance and visualized using Pymol (Alexander et al. 2011).

### Cell survival by MTT assay

2000 cells were seeded in replicate in a 96 well plate, exposed to conditioned media (CM) and incubated with MTT (0.5 mg/ml) reagent for 1.5 h. Media was removed, then crystals were dissolved in DMSO before absorbance was read at 570 nm.

### Immunofluorescence (IF)

The cells were seeded and stimulated with differentiation factors in an IF chamber (Fisher Scientific). For IF, the incubation chamber was removed and the slide, with the cells, was washed 3 times with PBS, followed by (1) cells fixation for 30 min at room temperature (RT) in 2% buffered formalin in PBS and (2) 3 washes (5 min each with PBS). The cells were then permeabilized with 0.1% Triton in TBS for 30 min followed by blocking for 1 h at RT using Donkey-serum IF buffer (10 mM Tris pH 7.4, 0.1 M MgCl2, 0.5% Tween20, 2% BSA, 5% donkey Sera). The cells were stained with primary antibodies (1:100) in blocking buffer (diluted in TBS, 1:1 ratio) overnight at 4 degrees Celsius. The next day, the cells were washed 3 times (5 min each) with 0.05% Triton in TBS before being stained with 2° antibodies (Alexa-fluor dye, Invitrogen; 1:2000) and Hoescht nuclear dye (1:2000) in 0.05% Triton-TBS for 1 h at RT on a shaker. Lastly, the slide was washed 4 times (10 min each) in 0.05% Triton-TBS before being mounted and visualized under fluorescence microscope. SHP2 antibodies used were raised in mouse (cat# LF-MA0186, Thermo Fisher) while PDHA1 antibodies (cat# 3205, cell signaling) were from rabbit origin.

### ROS assay by nitroblue tetrazolium (NBT) and dihydroethidium (DHE)

NBT (cat# N6876, Sigma) is a yellow powder which, in the presence of ROS, gets reduced to dark-blue crystals, therefore allowing for a visualization of ROS. For the assay (Furukawa et al. 2004), cells were incubated with 0.2% NBT in PBS for 1.5 h and switched to PBS. The crystals were visualized and imaged under the microscope, then dissolved in acetic acid (50%) and the absorbance was subsequently read at 560 nm. DHE assay was performed according to the manufacturer’s protocol (Abcam, cat# ab236206).

### Glycerol assay

For glycerol release, 23-day mature adipocytes were treated in phenol red-free serum-free media. The media was collected and incubated with assay reagents according to the manufacture’s protocol (Neogen, cat# K-GCROL).

### Lactate assay

This assay has been previously described (Manley et al. 2022). In brief, cells were grown in 96 well pate and the media were collected for measurement of secreted lactate according to the manufacturer protocol (Cayman Chemical; cat# 600,450).

### Transwell migration and invasion assays

PDAC cells (50 K) were seeded in the upper chamber (24-well inserts, 8 µm pore size; Corning Incorporated cat# 3422) of the transwell in serum-free media. 600 µL of conditioned media were pipetted into the bottom chamber of the plate and made a contact with the bottom of the insert. The insert was coated (invasion) or not (migration) with Matrigel and allowed to solidify in the incubator before the cells were seeded. Following the assay, cotton-tipped applicator was used to remove cells, from upper surface of the transwell, that did not migrate/invade. Then the migrated or invader cells were first fixed in 70% ethanol and stained with 0.2% crystal violet for 10 min each. The staining was dissolved in 50% acetic acid and absorbance was read at 590 nm.

### Cell cycle analysis

250 K cells were seeded in 6-well plate, synchronized in serum-free media for 24 h and then exposed to conditioned media for 18 h. This was followed by Propidium Iodide (PI) staining protocol, including incubation with RNAse, and cell cycle phases were evaluated by flow cytometry. The analysis was performed using the FlowJo software. The percentage of cells in each phase of the cell cycle were determined by PI intensity and quantified.

### ELISA

For the quantitative murine IL-6 assay, the Quantikine ELISA immunoassay kit (R & D System, cat# M6000B) was used. In brief, following treatment of the 23-day differentiated adipocytes in serum-free, the media was collected and used for the assay according to the manufacturer’s protocol.

### IL-6 neutralization

The levels of IL-6 in the media were first determined by quantitative ELISA as described above. The media was then incubated with IL-6 antibodies (cat# 16706181; Thermo Scientific, 50 ng/mL) overnight and used to grow PDAC cells; IgG antibodies were used as control.

#### RNA isolation and qPCR

Protocols have been previously described (Messaggio et al. 2017). The qPCR program was according to the three-step method in the protocol from the master mix vendor (APExBIO) and was as follow: initial denaturation (hold; 1 cycle): 95 °C for 2 min; 40 cycles of denaturation (95 °C for 15 s), annealing (50–60 °C for 30 s) and extension (72 °C for 30 s) followed by 1 cycle of 95 °C for 15 s, 60 °C for 1 min and 95 °C for 15 s. The primers used (below) are specific to mouse species:

PDHA1 forward: TGATCCGCCTTTAGCTCCATC; Reverse: TGTGACCTTCATCGGCTAGAA.

Ptpn11 (SHP2): cat# QT00103362, Qiagen.

### 2-NBDG (2-deoxy-2-[(7-nitro-2,1,3-benzoxadiazol-4-yl) amino]-D-glucose) uptake

For 2NBDG uptake assay, adipocytes were differentiated in a 96-well plate, then glucose and serum starved for 40 min prior to incubation with 2NBDG (200 µM, Cayman Chemical, Cat# 11046) in PBS for 80 min. At endpoint, cells were washed with PBS and 2NBDG content was measured via plate reading at excitation 475 nm, emission 550.

### Lentiviral transfection for SHP2 knockdown

For the generation of SHP2 knockdown cells, short hairpin RNA (shRNA) constructs, with scrambled SCR or targeting two independent regions of *SHP2* mRNA, were obtained from Millipore Sigma (Burlington, MA USA). These constructs were used to generate packaged lentiviruses by transfecting the constructs with packaging constructs (pCMV-dR8.2 #8455 and pCMV-VSV-G #8454, addgene) into HEK293T cells to produce viral supernatants which were subsequently used to transduce indicated cells. Successfully transfected cells were selected with puromycin treatment (2 µg/mL) until complete cell death was evident in un-infected cells.

#### Diet-induced obesity in mice

Methods have been previously described (Mendonsa et al. 2015). In brief, the mice were subjected to either a standard chow diet or high fat diet (TD88137 Teklad, 42% calories from fat, 42.7% from carbohydrates, and 15.2% from protein) for three months at which time the body weight was determined and tissues collected. Animals were handled in accordance with the Institutional Animal Care and Use Committee (IACUC) of the University of Kansas Medical Center.

### Patient samples

Adipose tissue samples were obtained from de-identified pre-operatively consented patients at the University of Kansas Medical Center via the Biospecimen Repository. The patient’s body mass index (BMI) was across the spectrum and categorized as follows: normal (18–24), overweight (25–29) and obese (over 30).

### Statistical analysis

For assessment of statistical significance, Ordinary one-way analysis of variance (ANOVA) was performed with Sidak’s multiple comparisons test/Dunnett’s multiple comparisons test, or where there are just two sample groups, a *t*-test was performed. The tests were performed using GraphPad Prism9 software. *p* < 0.05 was considered significant.

## Results

### Obesity harbors high levels of phospho-SHP2 and PDHA1 in the adipose tissue

**Fig. 1:**
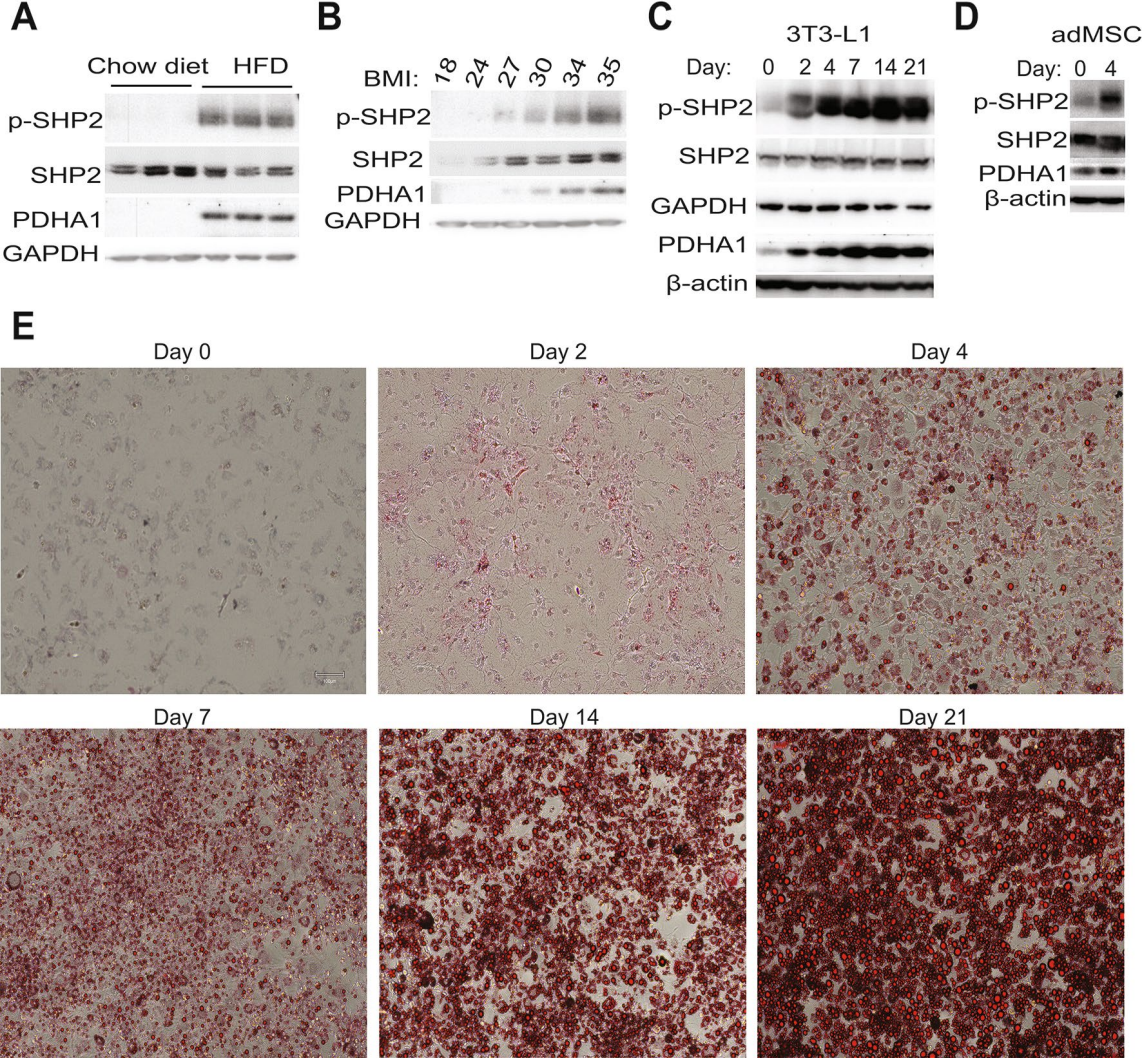
Obesity harbors high levels of phospho-SHP2 and PDHA1. **A**, **B** Lysates were prepared from white perigonadal adipose tissue from lean or obese mice (A) as well as from human patient white adipose samples (B). Equal amount of protein was resolved on SDS-PAGE followed by western blotting with indicated antibodies. **C**, **D** Indicated cells were stimulated with differentiation media as described in the methods and materials section. Cell lysate was prepared at indicated time points followed by western blotting with indicated antibodies (*n* = 3 experiments for C and *n* = 2 experiments for D). **E** 3T3-L1 cells were stained with Oil Red O (5 mg/ml) at indicated time points after induction of differentiation and then visualized under fluorescence microscope. Scale bar is 100 µm; *n* = 3 experiments

A loss-of-function mutation in SHP2 has been reported to slow down high fat diet-induced obesity in mice (Tajan et al. 2014). To begin to assess the contribution of SHP2 and PDHA1 to adipocyte development, we first induced obesity in a cohort of mice using a high fat diet (HFD) and maintained a cohort of lean mice on regular chow diet. The adipose tissue was harvested from the obese and lean mice and assessed for their level of the active (phosphorylated) form of SHP2 (p-SHP2). Recruitment of SHP2 to sites of activity and its activation has been shown to correspond to phosphorylation at Y542 (Feng et al. 1993; Vogel et al. 1993; Lu et al. 2003). High levels of p-SHP2 (Y542) were detected in the adipose tissue of obese mice (Fig. 1A). The level of PDHA1, the rate-limiting component of the mitochondrial pyruvate dehydrogenase complex (PDHc), was also high in the obese adipose samples (Fig. 1A). Next, in a correlative analysis, we examined adipose tissue samples from human obese patients and found that both p-SHP2 and PDHA1 levels were increased in the adipose tissue as the BMI increased (Fig. 1B). To study the kinetics of p-SHP2 and PDHA1 accumulation, we first initiated adipogenic differentiation in the commonly used adipocyte precursor cells, 3T3-L1. Analysis of p-SHP2 and PDHA1 levels demonstrated a time-dependent increase after onset of differentiation (Fig. 1C). To confirm the results above, we also stimulated human adipose-derived mesenchymal stem cells (adMSC), with adipogenic factors. Here too, p-SHP2 and PDHA1 levels increased after initiation of differentiation (Fig. 1D). To further validate the observations, we isolated pre-adipocytes within the stromal vascular (SV) fraction from freshly isolated lean mouse adipose tissues (Aune et al. 2013). Adipogenic stimulation of these cells also increased p-SHP2 and PDHA1 (Supplementary Fig. 1A). Finally, assessment of lipid accumulation with Oil red O staining demonstrated that the increase in p-SHP2 and PDHA1 levels correlated with a time-dependent increased neutral lipid accumulation in the maturing adipocytes (Fig. 1E).

### SHP2 regulates PDHA1 protein levels

**Fig. 2:**
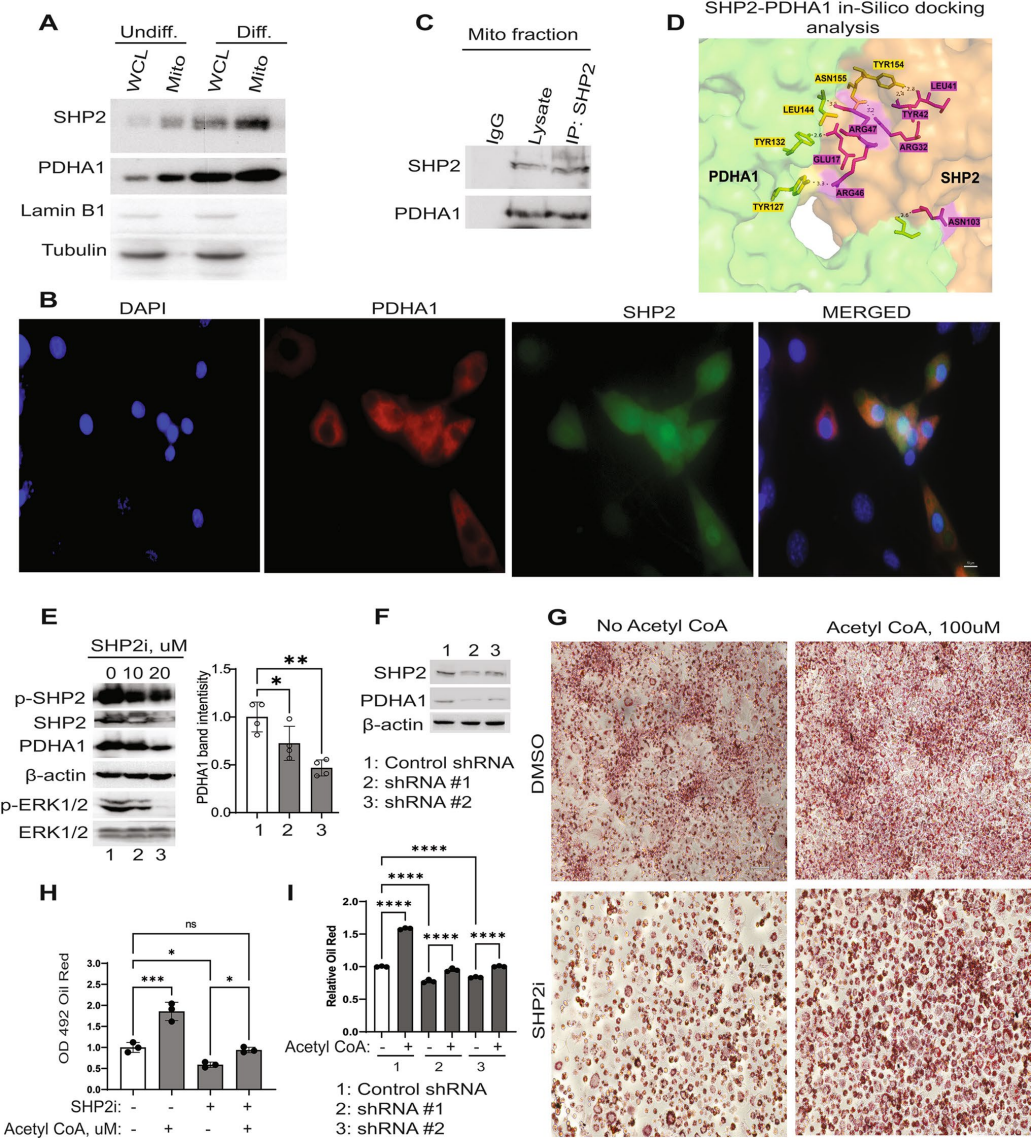
SHP2 regulates PDHA1 protein levels. **A** Control undifferentiated (Undiff.) and 7 days differentiated (Diff.) 3T3-L1 were assessed for indicated proteins in whole cell lysates (WCL) or isolated mitochondria (Mito). The purity of the mitochondrial fraction was evaluated with antibodies against mitochondrial protein (PDHA1), cytoplasmic protein (Tubulin) and nuclear protein (Lamin B1). **B** Immunofluorescence assay to assess localization of SHP2 (green) and PDHA1 (red) in the differentiated 3T3-L1. DAPI (blue) was used as a nuclear counterstain; scale bar is 10 µm. **C** Immunoprecipitation (IP) assay of SHP2 from differentiated adipocyte mitochondria to assess interaction with PDHA1. **D** In-silico modeling of SHP2-PDHA1 interaction, showing the predicted interacting amino acids in stick format and their respective bond distances. The interaction of SHP2 with PDHA1 was analyzed using the crystal structures of the proteins and the GrammX online server. The most favorable prediction was selected, and the interaction was studied using Pymol. Color scheme-Green: PDHA1 protein, Orange: SHP2 protein, Yellow: interacting amino acids of PDHA1, Pink: interfacing amino acids of SHP2. **E** 3T3-L1 cells were stimulated with differentiation media containing indicated concentrations of SHP2i, or DMSO control (0), followed by western blotting with indicated antibodies; *n* = 4. **F** SHP2 gene was knocked down in 3T3-L1, by shRNA, followed by induction of differentiation for 6 days. Indicated proteins were assayed by western blot. **G-H** 3T3-L1 cells were stimulated in the presence or absence of SHP2 inhibitor with or without supplementation with acetyl CoA (100 µM) for 5 days; media replenished every 2 days. The adipocytes were subsequently stained with Oil Red O to visualize lipid content. For quantification, the retained dye was dissolved in 100% isopropanol and absorbance read at 492 nm; **I** SHP2 knock down and their control cells were stimulated with adipogenic factors with/without acetyl CoA as in F-G; *n* = 3, error bars, SD; *p < 0.05; scale bar is 100 µm

We have previously observed that glucose-deprived pre-adipocytes (Supplementary Fig. 1B) and non-adipocyte precursor cells alike (Supplementary Fig. 1C), induced SHP2 phosphorylation. This suggested a crucial conserved role for SHP2 in sensing nutrient levels, such as glucose, in cells. It was reported that a deletion of SHP2 in lean mouse adipose tissue led to glucose dysregulation and lipodystrophy (He et al. 2013). Additional studies implicated SHP2 in energy metabolism (Zhang et al. 2004; He et al. 2013). We reasoned that the increase in p-SHP2 and PDHA1 in obese conditions (Fig. 1) is a response to a change in metabolic demand which requires SHP2 and PDHA1 activity. Assessment of proteins involved in glucose metabolism demonstrated an induction of the expression of glucose transporter type 1 (Glut1) and Hexokinase (HK) in the differentiating adipocytes (Supplementary Fig. 1D). Moreover, lactate secretion increased (Supplementary Fig. 1E). While HKs are the rate-limiting enzyme for glycolysis, PDHA1 can be rationalized as a potential molecular node between glycolysis, ATP generation (Sun et al. 2015; Sheeran et al. 2019) and perhaps other anaplerotic pathways such as lipogenesis. We assessed whether SHP2 affects adipocyte metabolism through PDHA1. First, we isolated the mitochondria from 3T3-L1 cells stimulated or not with adipogenic factors and found SHP2 present in the mitochondrial fractions (Fig. 2A). Immunofluorescent detection also showed that while SHP2 had diffuse cellular staining, including nuclear staining, some SHP2 also co-localized with PDHA1 (Fig. 2B). Immunoprecipitation (IP) of SHP2 from mitochondrial fraction from differentiated adipocytes followed by western blotting with PDHA1 antibodies suggests that SHP2 and PDHA1 are in the same complex and may directly interact (Fig. 2C). Further, in-silico modeling of protein–protein interaction, using the crystal structures of human SHP2 and PDHA1, predicts a direct interaction of SHP2 with PDHA1 via the SH2 domain of SHP2 (Fig. 2D; Supplementary Fig. 1F). To determine whether SHP2 regulates PDHA1 protein levels, we first suppressed SHP2 activity in the differentiating cells by pharmacological means, using the specific inhibitor, SHP099. SHP2 inhibition corresponded with a reduced PDHA1 level upon induction of differentiation (Fig. 2E); inhibition of SHP2 via SHP099 was validated by suppression of downstream p-ERK (Fig. 2E). In other cellular and biological contexts, adipocyte conditioned media (CM)-mediated induction of PDHA1 in pancreatic cancer cells appears to also be suppressed after SHP099 treatment (Supplementary Fig. 1G). To further validate these results, we also knocked down SHP2 in the pre-adipocytes and induced differentiation. Evaluation of PDHA1 level also showed a reduced PDHA1 expression (Fig. 2F). While PDHA1 gene transcript levels increased after induction of differentiation (Supplementary Fig. 1H), the expression levels were not significantly affected by SHP2 inhibition (Supplementary Fig. 1I–J). SHP2 inhibition hampered lipid accumulation in the differentiating adipocytes. However, that effect was partially rescued by supplementation with Acetyl Coenzyme A, the downstream product of PDHA1 activity (Fig. 2G–I), suggesting a functional link of SHP2 to PDHA1 activity.

### SHP2 promotes reactive oxygen species (ROS)-driven adipogenesis

**Fig. 3:**
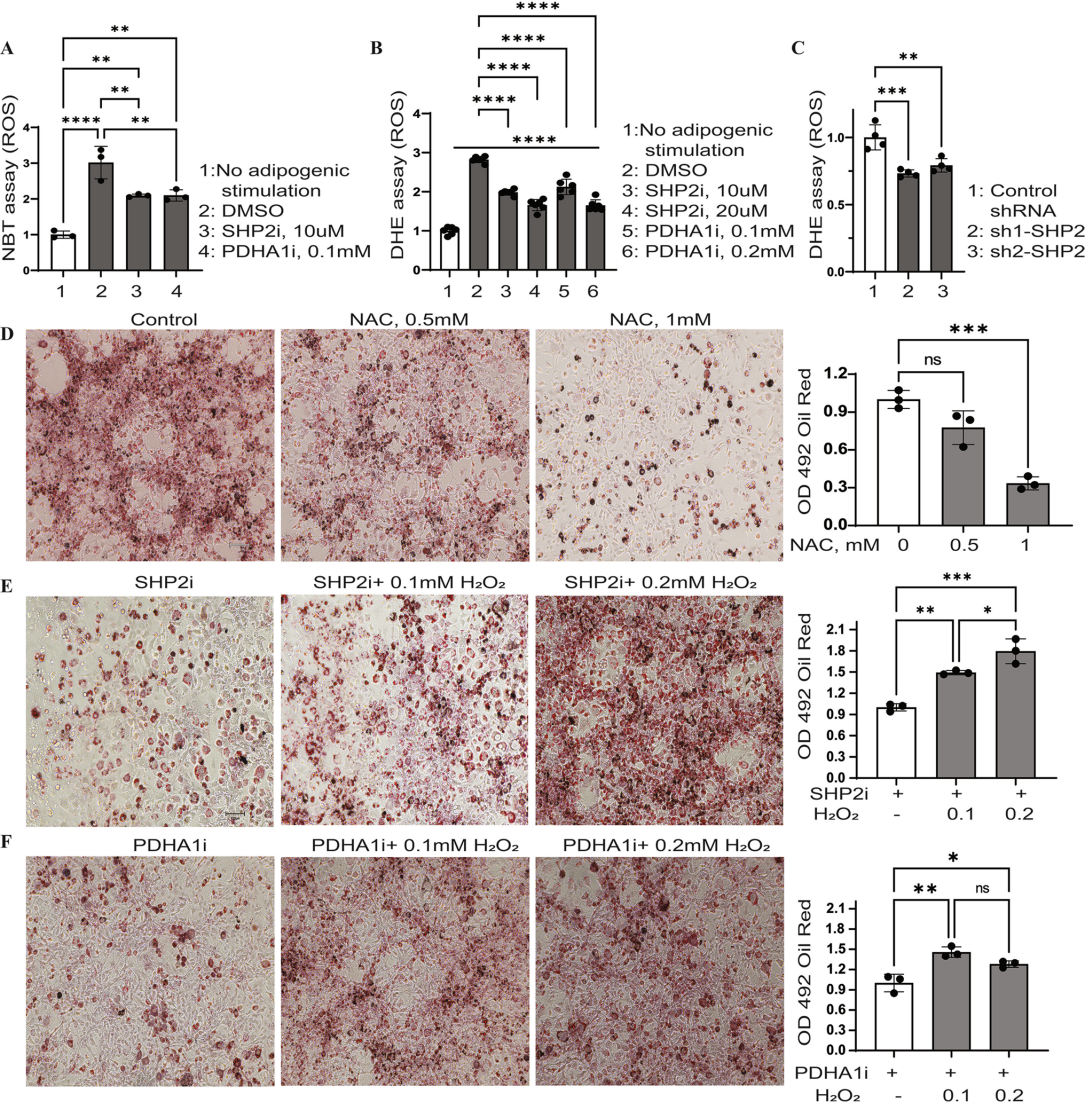
SHP2 promotes reactive oxygen species (ROS)-driven adipogenesis. **A** 3T3-L1 cells were subjected to differentiation for 5 days in the presence/absence of SHP2 inhibitor (SHP2i, 10 µM), PHDA inhibitor (PDHA1i, 0.1 mM), or DMSO (vehicle control). The cells were then stained with 0.2% Nitroblue Tetrazolium (NBT) in PBS for 1.5 h to assess ROS levels. After microscopic visualization, the NBT crystals were dissolved in acetic acid (50%) and the absorbance was read at 560 nm (Graph bottom left; *n* = 3; error bars, SD; *p < 0.05). **B** Similar to A except that ROS was assessed by DHE. **C** SHP2 knock down 3T3-L1 cells were differentiated and assayed for ROS by DHE. **D–F** 3T3 cells were stimulated in the presence/absence of N-acetyl cysteine (NAC, **D**), SHP2i (10 µM, **E**), or PDHA1i (0.1 mM, **F**) with/without H_2_O_2_ (**E**, **F**) for 5 days. The cells were stained with oil red O as described above and absorbance read at 492. Graphs next to each panel; *n* = 3; error bars, SD; *p < 0.05. scale bar is 100 µm

It was reported that treatment of adipose-derived stem cells with ROS precursor H_2_O_2_ was sufficient to induce intracellular accumulation of oil droplets (Higuchi et al. 2013), which emphasizes the positive role of ROS in adipogenesis. As a mitochondrial protein, PDHA1 is key for mitochondrial activity. The latter is widely thought as the main source of ROS generation in cells (Li et al. 2013). Thus, we tested whether SHP2 activity would also affect ROS. Assessment of ROS levels, by Nitroblue Tetrazolium (NBT) reduction into dark-blue crystal (Furukawa et al. 2004), showed that ROS generation did increase with differentiation but was diminished with SHP2i (Fig. 3A); inhibition of PDHA1 also produced similar results (Fig. 3A). To further evaluate these effects, we used a Dehydroethidium (DHE) assay to measure ROS. SHP2 inhibition or knockdown suppressed ROS generation by DHE assay (Fig. 3B, C). Supplementation of the differentiating media with H_2_O_2_ modestly enhanced lipid accumulation (Supplementary Fig. 2A), whereas addition of ROS scavenger, N-acetyl cysteine (NAC), impaired lipid accumulation (Fig. 3D). Given that SHP2i/PDHA1i-treated cells exhibited significantly low endogenous ROS levels (Fig. 3A–C), their differentiation media was supplemented with H_2_O_2_ which rescued lipogenesis in those cells although to a lesser degree in PDHA1i-treated cells (Fig. 3E, F). Together, these results support the idea that SHP2 promotes ROS-driven adipogenesis.

### ROS generation depends on SHP2-mediated glucose uptake

**Fig. 4:**
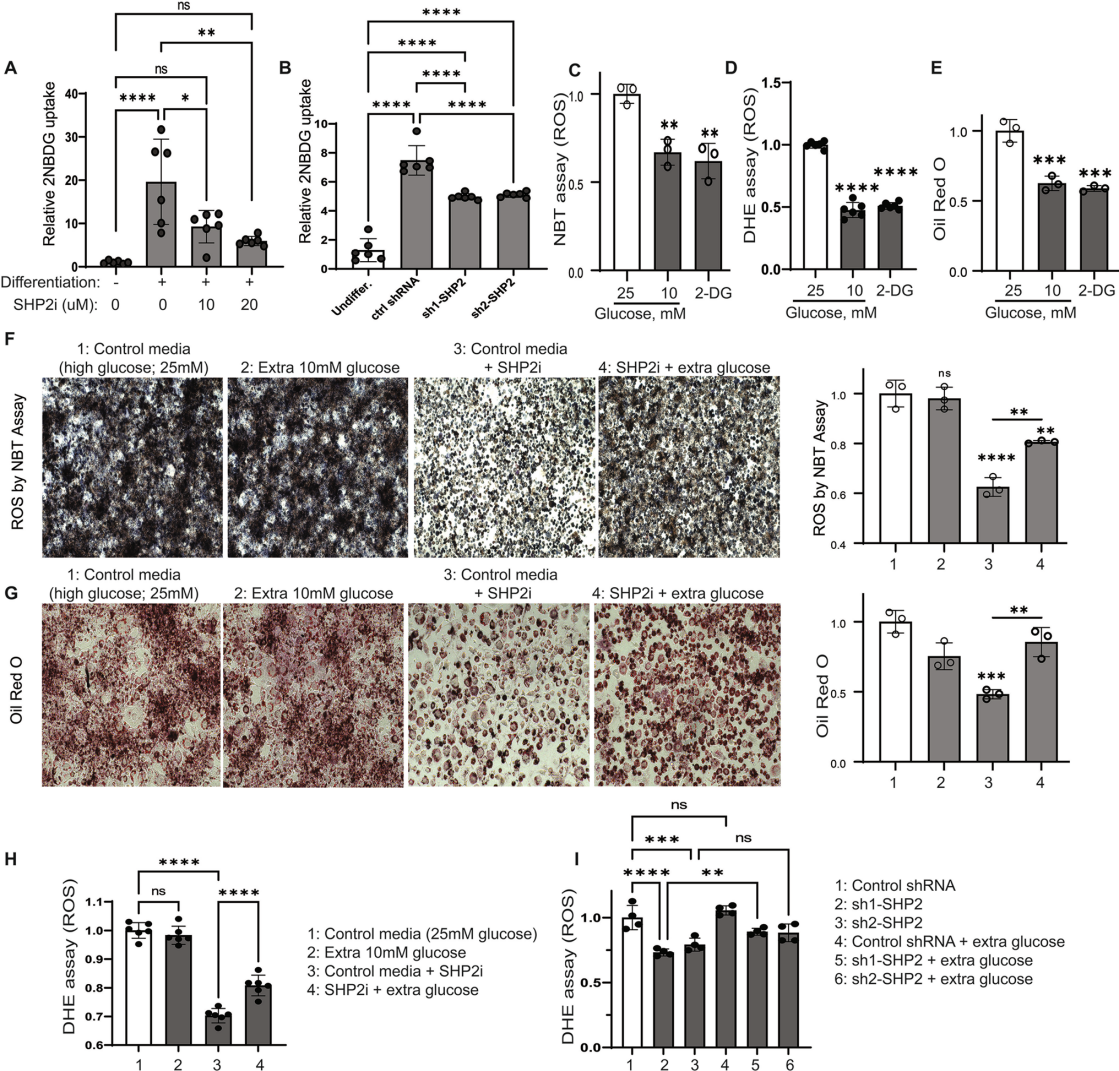
ROS generation depends on SHP2-mediated Glucose uptake. **A** 3T3 cells were differentiated with SHP2i for 6 days and then glucose and serum-starved for 40 min prior to incubation with 2NBDG (300 µM) for 80 min. At the end points, the cells were washed and assessed for 2NBDG content by plate reading (excitation 475 nm; emission 550 nm). **B** Similar to A except that SHP2 knock down cells were used and 200 µM of 2NBDG was utilized**. C–G** Cells were differentiated with normal differentiation media (25 mM of glucose) containing 2-Deoxy-D-Glucose (2DG) or in low glucose media (10 mM) or containing SHP2i (10 µM, **F**–**G**) with/without extra glucose (10 mM, **F**–**G**) for 6 days at which time the cells were assayed for lipid content or ROS as described in Fig. 3. *n* = 3; error bars, SD; *p < 0.05. *A*, *N* = 6. **H** Similar to F except that DHE assay was used to measure ROS. **I** Similar to H except that the assay was run on SHP2 knock down cells; the experiment was done simultaneously with 3C and the panel (I) uses the same sh1-SHP2 and sh2-SHP2 groups as 3C

Based on the results above and other observations that low cellular glucose levels activate SHP2 (Supplementary Fig. 1B, C), we next tested whether SHP2 links ROS to adipogenesis via cellular glucose utilization. Glucose uptake assay, by 2NBDG uptake, indicated that cells exposed to differentiation stimuli increase their intracellular glucose levels (Fig. 4A, B). However, this glucose uptake was reduced in the presence of a SHP2 inhibitor (Fig. 4A) or with SHP2 knockdown (Fig. 4B); SHP2 inhibition after differentiation also decreased glucose uptake (Supplementary Fig. 2B). When glucose levels were lowered in the differentiation milieu or a non-metabolizable glucose analogue, 2-Deoxy-glucose, was added, ROS generation decreased (Fig. 4C, D), and lipid accrual was also impaired in the differentiating cells (Fig. 4E). Likewise, SHP2 inhibition or knockdown not only led to a decrease in glucose uptake (Fig. 4A, B), but also diminished ROS levels (Fig. 3A–C and 4F) and impaired lipid accumulation (Fig. 4G), which mimicked the effects of low glucose (Fig. 4C–E). Supplementation of SHP2 inhibitor-treated or SHP2 knocked down cells with glucose rescued ROS generation (Fig. 4H–I) and lipid content in the cells (Fig. 4G), suggesting that SHP2 promotes ROS-driven adipogenesis via regulation of glucose.

### SHP2 inhibition or ROS scavenging in 23-day mature adipocytes evokes a molecular signature of lipolysis and thermogenesis

**Fig. 5:**
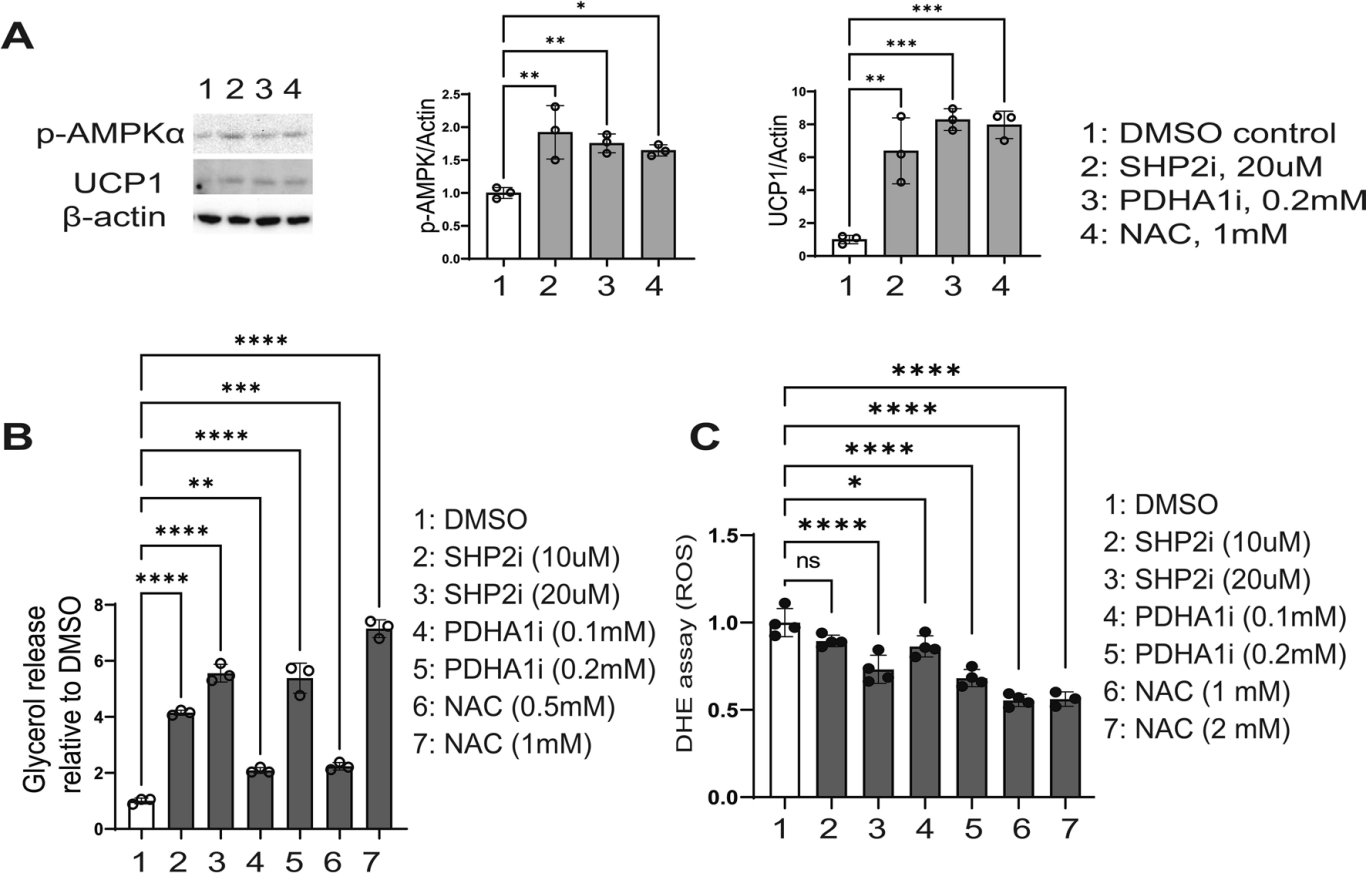
SHP2 inhibition in fully mature adipocytes evokes molecular signature of lipolysis and thermogenesis. **A** 3T3-L1 cells were differentiated and maintained to fully mature adipocytes, until day 23, before being treated with indicated inhibitors for 96 h. Indicated proteins were assessed by western blot; to the right-hand side is the quantification of the bands by densitometry. **B** 23-day mature adipocytes were treated with indicated inhibitors for 96 h in phenol red-free serum-free media. The media was then collected and used for the glycerol release assay according to the manufacturer’s protocol. **C** 23-day mature adipocytes were treated as in (A) and assayed for ROS by DHE assay; *n* = 3 for A–C; error bars, SD; *p < 0.05

The results above demonstrated that SHP2 and PDHA1 activities are critical for initiation and progression of adipogenesis. They also implied that their activity is crucial for lipid stability post-adipogenesis. To investigate the role of SHP2 and PDHA1 in adipocyte maintenance, we differentiated pre-adipocytes into fully mature adipocytes and then exposed them to respective inhibitors. Perilipin-1 is a lipid droplet stabilizer and a marker for adipogenesis (Kern et al. 2004), which we found to accumulate in differentiating adipocytes in a time dependent manner until day 14, after which the levels appears to plateau (Supplementary Fig. 2C). These results indicate that the adipocytes don’t reach their full lipid stability until at least day 14 post initiation of differentiation. Accordingly, we let the differentiated adipocytes mature past 14 days (at day 23) and then treated them with a SHP2 or PDHA1 or ROS inhibitor. Though, visually, we did not observe an appreciable difference in the lipid droplets size with SHP2i treatment, the treatment induced expression of pro-lipolytic proteins, p-AMPKα (Kim et al. 2016) (Fig. 5A). Additionally, p-HSL (a lipase) appeared to increase but did not yield a clean band (Supplementary Fig. 2D) which precluded a quantitative evaluation. Importantly, these effects also corresponded to an induction of the thermogenic protein Uncoupling Protein 1 (UCP1) (Fig. 5A). Similar results were obtained with PDHA1 inhibitor and ROS scavenger N-acetyl cysteine, NAC (Fig. 5A). Assessment of the release of glycerol, the by-product of triglyceride breakdown commonly used as a marker of lipolysis (Nielsen et al. 2014), indicated that loss of SHP2 or PDHA1 activity or ROS scavenging in the mature adipocytes induces a significant release of glycerol (Fig. 5B). NAC treatment decreased ROS levels but not as dramatically as expected for a ROS scavenger (Fig. 5C), suggesting that the obese adipocytes may be particularly resistant to anti-ROS intervention. Likewise, SHP2i or PDHA1i treatment reduces ROS levels in mature adipocytes but not dramatically (Fig. 5C).

### Adipocytes with SHP2 or PDHA1 inhibition failed to drive the growth of pancreatic cancer cells

**Fig. 6:**
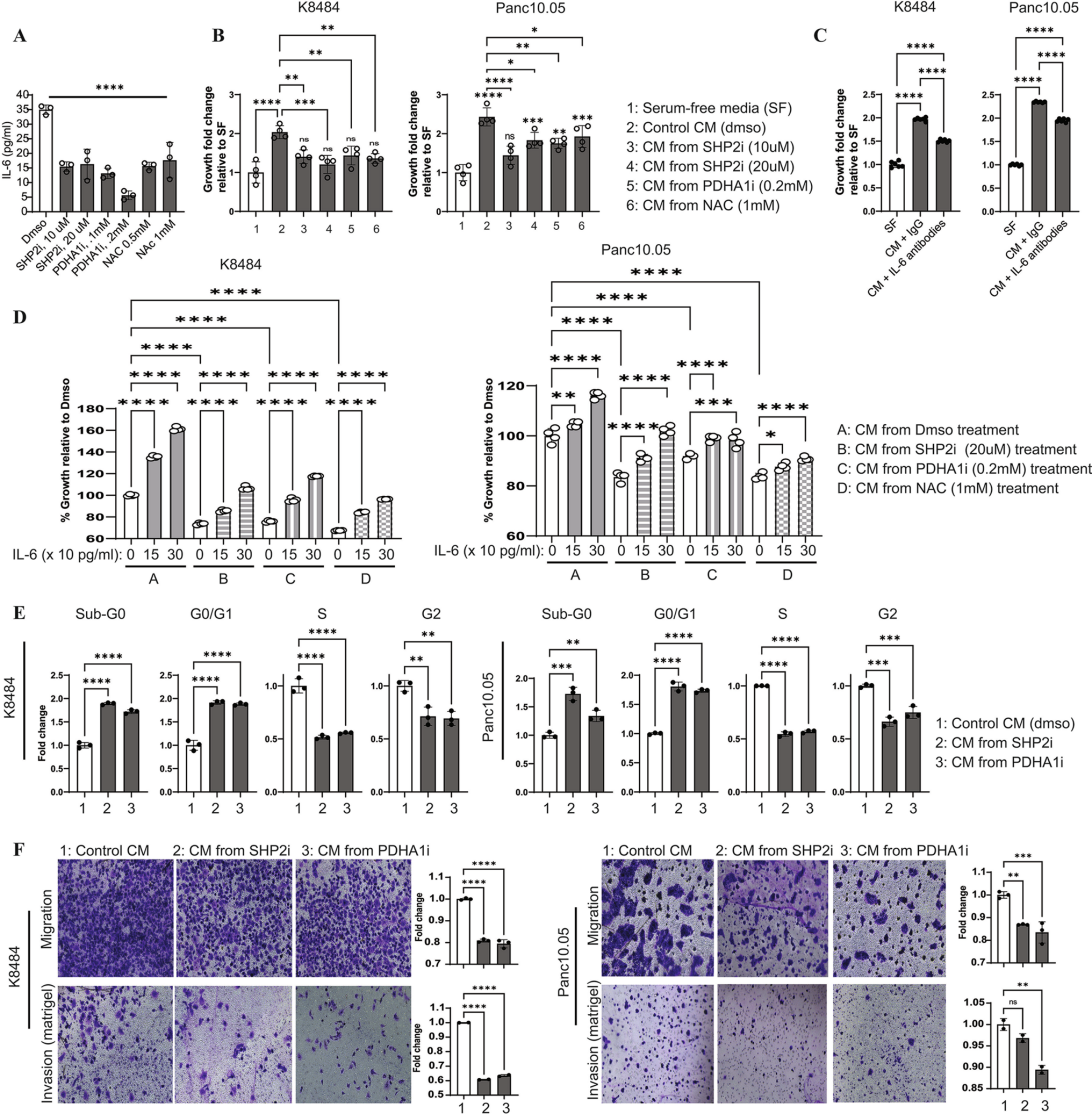
Adipocytes differentiated in the presence of SHP2 or PDHA inhibitor failed to drive pancreatic cancer hallmarks. **A** 23-day mature adipocytes were treated with indicated inhibitors in serum-free media for 96 h; media was collected and used to quantitatively assay mouse IL-6 according to the manufacture’s protocol; *n* = 3, **** is the comparison of Dmso to each treatment condition. **B** After treating the 23-day mature adipocytes with the indicated inhibitors, conditioned media (CM) was collected in serum-free media and applied to indicated PDAC cell lines for 48 h and an MTT assay was performed; *n* = 4. **C** IL-6 neutralization partially suppresses CM-mediated PDAC growth. IL-6 was neutralized in the CM used in (B) and applied to indicated cells for 48 h followed by MTT assay. **D** Indicated cells were cultured in indicated CM as described in (B) with/without recombinant murine IL-6 for 48 h followed by MTT assay; *n* = 4, error bars, SD; *p < 0.05. **E–F** SHP2 or PDHA1 inhibition in mature adipocyte alters PDAC cell cycling (E) or migration and invasion (**F** when exposed to CM from the respective adipocytes. Indicated cells were either synchronized in serum-free media for 24 h followed by stimulation with indicated CMs for 18 h and assessment of cell cycle phases by flow cytometry (E); or the cells were seeded in serum-free media in the transwell upper chamber (8-micron pore size) coated (invasion) or not (migration) with Matrigel and exposed to indicated CMs in the lower chamber for 18 h. This was followed by crystal violet staining (0.2%) of cells on the other side of the membrane. After imaging the cells, the crystals were dissolved in 50% acetic acid and absorbance was read at 590 nm

Obesity is a major risk factor for numerous cancers, including pancreatic ductal adenocarcinoma (PDAC) (Zyromski et al. 2009; Rebours et al. 2015). Obesity promotes PDAC through secretion of pro-tumorigenic cytokines by adipose tissue resident cells including adipocytes, one which is interleukin-6 (IL-6) (Path et al. 2001; Wueest and Konrad 2018; Han et al. 2020). IL-6 has emerged as a key mediator of PDAC cells migration (Nagathihalli et al. 2016; Razidlo et al. 2018; Thomas 2019) and obesity-dependent cancer development (Park et al. 2010). Given that adipocytes are sufficient to drive tumor progression (Nieman et al. 2011; Huang et al. 2017; Wu et al. 2019), we sought to assess the relevance of SHP2/PDHA/ROS to adipocyte-driven cellular hallmarks of PDAC. We assessed the contribution of SHP2/PDHA/ROS to IL-6 secretion in adipocytes by treating 23-day mature adipocytes with inhibitors for SHP2, PDHA1, or ROS and subsequently collecting the media for quantitative cytokine assessment. Inhibition of SHP2, PDHA1, or ROS decreased the levels of secreted IL-6 (Fig. 6A) compared to a DMSO treatment control. Post analysis, we utilized the adipocyte conditioned media (ACM) to functionally test the response of PDAC cell lines. PDAC cells demonstrated increased growth when cultured in the ACM from control DMSO-treated adipocytes (Fig. 6B). However, PDAC cells cultured in ACM from adipocytes treated with inhibitors exhibited a decreased growth (Fig. 6B). Neutralization of IL-6 in the control DMSO-treated ACM suppresses growth, although not dramatically (Fig. 6C), suggesting that other factors besides IL-6 might also be driving the ACM-mediated PDAC cells growth. Conversely, supplementation of murine IL-6 to the respective ACMs enhances the growth of PDAC cells (Fig. 6D). These results suggest that regulation of the SHP2-PDHA1-ROS axis is critical for adipocyte-mediated influence of PDAC cellular function. To expand on that, the effect of the respective ACMs on various pancreatic cancer neoplastic features was evaluated. Assessment of how exposure of PDAC cells to the conditioned media from adipocytes treated with SHP2/PDHA1 inhibitor affects cell cycle demonstrates a slight increase in sub-G0, and G0/G1 but a decrease in S and G2 population when the ACMs are derived from adipocytes exposed to SHP2/PDHA1 inhibitors (Fig. 6E). Migration and invasion assays also show a slight suppression of PDAC cells migratory and invasive potential when exposed to conditioned media derived from adipocytes treated with SHP2 or PDHA1 inhibitors (Fig. 6F).

### PDHA1 activity is required for adipocyte-mediated pancreatic cancer growth

**Fig. 7:**
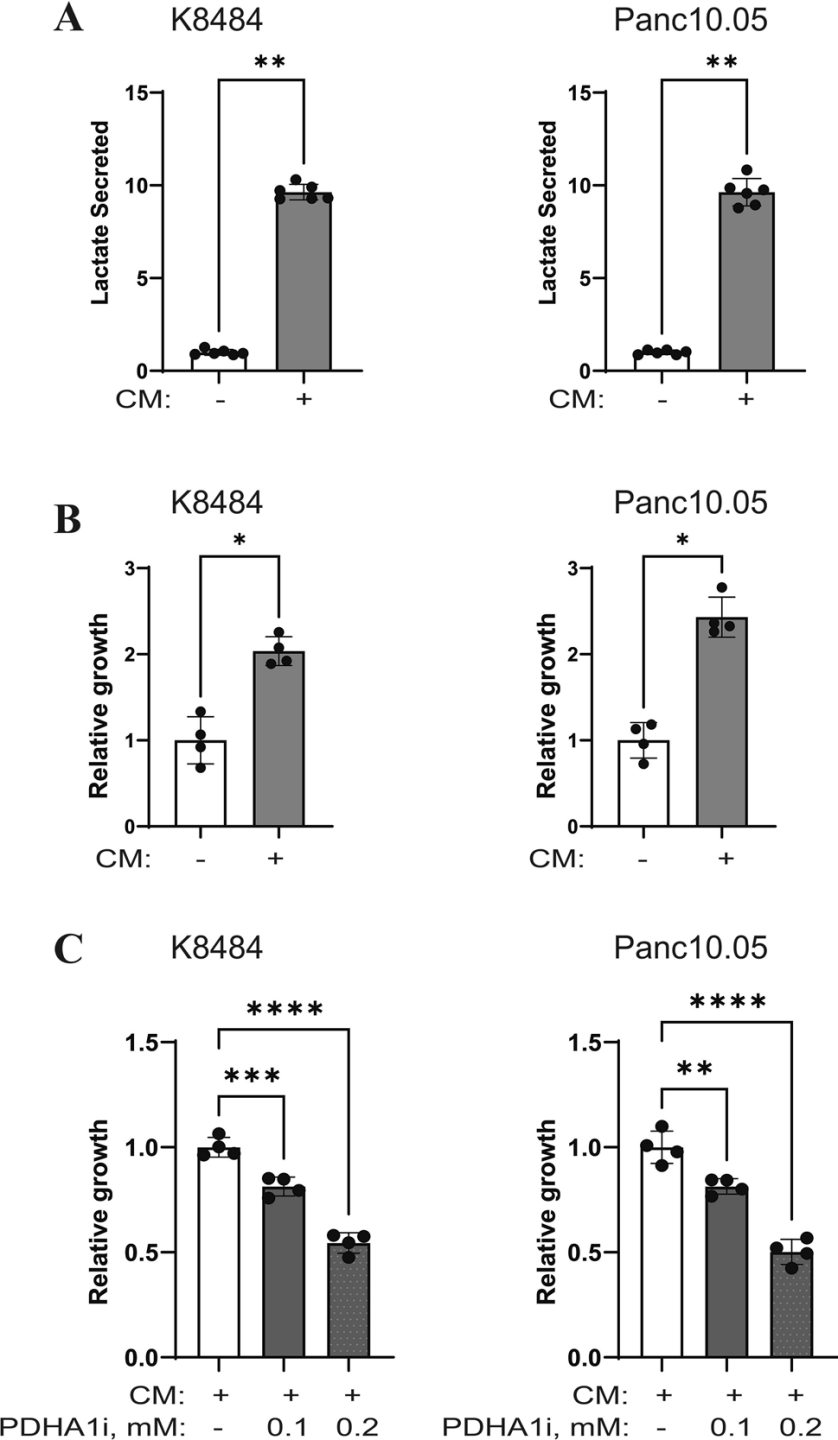
Adipocyte conditioned media alters PDAC cell metabolism and growth. **A** Indicated PDAC cells were grown in conditioned media (CM) from mature adipocytes for 48 then assayed for secreted lactate in the media. **B** This panel uses the same CM-treated group as in Fig. 6B. **C** Indicated PDAC cells were pre-treated with PDHA1 inhibitor for 1 h before being stimulated with adipocyte CM with/without PDHA1 inhibitor for 48 h followed by assessment of growth; *n* = 4, error bars, SD; *p < 0.05

PDHA1 is a key metabolic enzyme in the cell, coordinating various aspects of cellular metabolism (Sun et al. 2015; Chen et al. 2018; Sheeran et al. 2019; Cai et al. 2020). As described earlier, exposure of the PDAC cells to the adipocyte conditioned media increases PDHA1 levels (Supplementary Fig. 1G) and causes an increased lactate secretion by the PDAC cells (Fig. 7A). An increased lactate secretion is an indication of increased glucose utilization which could be a direct result of glucose preference or indirectly influenced by the presence/depletion of alternate carbon sources such as glutamine. The increased lactate secretion correlated with an increased growth of PDAC cells (Fig. 7B). To test the requirement of PDHA1 for growth in response to adipocyte-derived factors, PDAC cells were pre-treated with the PDHA1 inhibitor before their exposure to the conditioned media. PDHA1 inhibition resulted in a suppression of growth (Fig. 7C).

## Discussion

Obesity is associated with the development and progression of several cancers including pancreatic cancer (Zyromski et al. 2009; Aune et al. 2012; Genkinger et al. 2015). As the main cell component of the adipose tissue (Bae et al. 2012), adipocytes are conceptually central to understanding the relation between adipose tissue dysfunction and pancreatic cancer. While SHP2 has been implicated in adipocyte maturation (Uehara et al. 2007; Bae et al. 2012; He et al. 2013) and pancreatic cancer development (Ruess et al. 2018), the roles of SHP2 in adipocyte maintenance and adipocyte-cancer cell interaction has not been explored. Here, we demonstrate a SHP2-PDHA1-ROS regulatory axis in adipocyte maintenance with relevance to adipocyte-tumor crosstalk.

Our analysis has revealed that phospho-SHP2 and PDHA1 protein increased upon induction of adipogenesis (Fig. 1C–D, Supplementary Fig. 1A), correlating with induction of a protein signature for glycolysis (Supplementary Fig. 1D) and ROS generation (Fig. 3A, B). Through our investigation, we found that SHP2 localized to the mitochondria (Fig. 2A, B) during initiation of differentiation, where it localized with PDHA1 (Fig. 2A, B) and immunoprecipitated together (Fig. 2C). SHP2 inhibition or knock down decreases PDHA1 protein levels (Fig. 2E–F), indicating that the level of PDHA1 is dependent upon SHP2 activity. Although the mechanism is not clear yet, we speculate that SHP2 regulates PDHA1 post-translationally through protein–protein interaction (Fig. 2C, D; Supplementary Fig. 1F). Future studies would determine the biology of the amino acids identified by the in-silico analysis (Supplementary Fig. 1F), in cell metabolism. Based on (1) the induction of SHP2 phosphorylation in response to glucose deprivation and (2) glucose uptake regulating adipocyte maturation, it is possible that SHP2 might be regulated by a change in glucose demand in the developing adipocytes. Furthermore, the mechanism by which SHP2 translocates to the mitochondria would be interesting to investigate in future studies; previous studies showed that SHP2 mitochondrial localization is mediated through protein–protein interaction, such as with ANT1 protein (Guo et al. 2017).

The high levels of p-SHP2 and PDHA1 in obese adipose tissues (Fig. 1A, B) indicate important roles for SHP2 and PDHA1 in obesity initiation. Inhibition of SHP2, PDHA1, or ROS in fully mature adipocytes evokes a molecular signature of lipolysis and thermogenesis (Fig. 5A) which corresponded with lipolysis (Fig. 5B), suggesting critical roles of SHP2, PDHA1 and ROS in lipid stability. Previous studies have also implicated SHP2 in ROS production in other cell types (Wang et al. 2012; Xu et al. 2013; Li et al. 2015; Ghimire et al. 2019). Additional studies reported elevated levels of ROS in obese adipose tissue as well as in differentiating adipocytes (Furukawa et al. 2004). Our analysis indicates that SHP2 promotes a ROS-driven adipogenesis (Fig. 3).

As an energy (ATP) sensor, the induction of p-AMPKα with SHP2, PDHA1 or ROS inhibition in the mature adipocytes (Fig. 5A), correlating with the expression of the thermogenic protein uncoupling protein 1 (UCP1), suggests some form of energy dysregulation. While UCP1 is typically a brown adipocyte/adipose tissue marker, induction of UCP1 expression was also reported in white adipose tissues (inguinal) upon chemically-induced energy stress (Pfuhlmann et al. 2018). Furthermore, targeted expression of UCP1 in white adipose tissue increased thermogenesis and decreased adiposity (Zheng et al. 2017). Of note, inhibition of SHP2, PDHA1, or ROS in the mature adipocytes induces lipolysis and molecular markers for thermogenesis (Fig. 5A, B). These effects indicate that SHP2-PDHA1-ROS may mediate lipid energy storage. It is also possible that SHP2, PDHA1 and ROS help maintain white adipocytes by suppressing molecular signaling that controls the switch to brown adipocytes.

Adipocyte dysfunction plays an important role in adipocyte-tumor crosstalk and regulation of cancer progression. Mature adipocytes treated with SHP2, PDHA1, or ROS inhibitors failed to drive various pancreatic neoplastic features (Fig. 6B, E–F). Moreover, inhibition of SHP2, PDHA1, or ROS decreased the secretion of the pro-inflammation cytokine, IL-6 (Fig. 6A), a key mediator of obesity-mediated PDAC hallmarks (Park et al. 2010; Nagathihalli et al. 2016; Razidlo et al. 2018; Thomas 2019). This effect corresponds to a decrease in PDAC cells cycling, growth, migration, and invasion (Fig. 6B, E–F). Neutralization of IL-6 partially suppresses PDAC cell growth (Fig. 6C), suggesting that there might be other factors besides IL-6 driving the cancer-promoting molecular signals originating from the adipocytes. The decrease in IL-6 secretion by the adipocytes upon inhibition of SHP2, PDHA1 or ROS also suggests that the SHP2-PDHA1-ROS axis might regulate inflammation in the adipose tissue, and thereby indirectly influences cancer progression. Considering that other studies linked SHP2 activity to inflammation (Giri et al. 2012; Zhao et al. 2016; Hsu et al. 2017; Xiao et al. 2019; Xiao et al. 2020; Paccoud et al. 2021), future studies are warranted to investigate the role SHP2-PDHA1-ROS in regulation of stromal-derived inflammatory mediators which may or may not be specific to just adipocytes.

In conclusion, the SHP2-PDHA1-ROS axis plays an important role in adipocyte maintenance with a potential regulatory impact on cytokine production and pancreatic cancer cell growth. Inhibition of this axis may help alleviate increased adiposity and curb obesity-associated pro-tumorigenic signaling in the obese population.

## Supplementary Information

Below is the link to the electronic supplementary material.

Supplementary file1 (TIF 2177 kb)

Supplementary file2 (TIF 3427 kb)

Supplementary file3 (TIF 36 kb)
