# Supplementary figures and images for: SHP2 regulates adipose maintenance and adipocyte-pancreatic cancer cell crosstalk via PDHA1
Source: J Cell Commun Signal. 2022 Sep 8;17(3):575–90. doi: 10.1007/s12079-022-00691-1 (PMC10409927; doi:10.1007/s12079-022-00691-1)

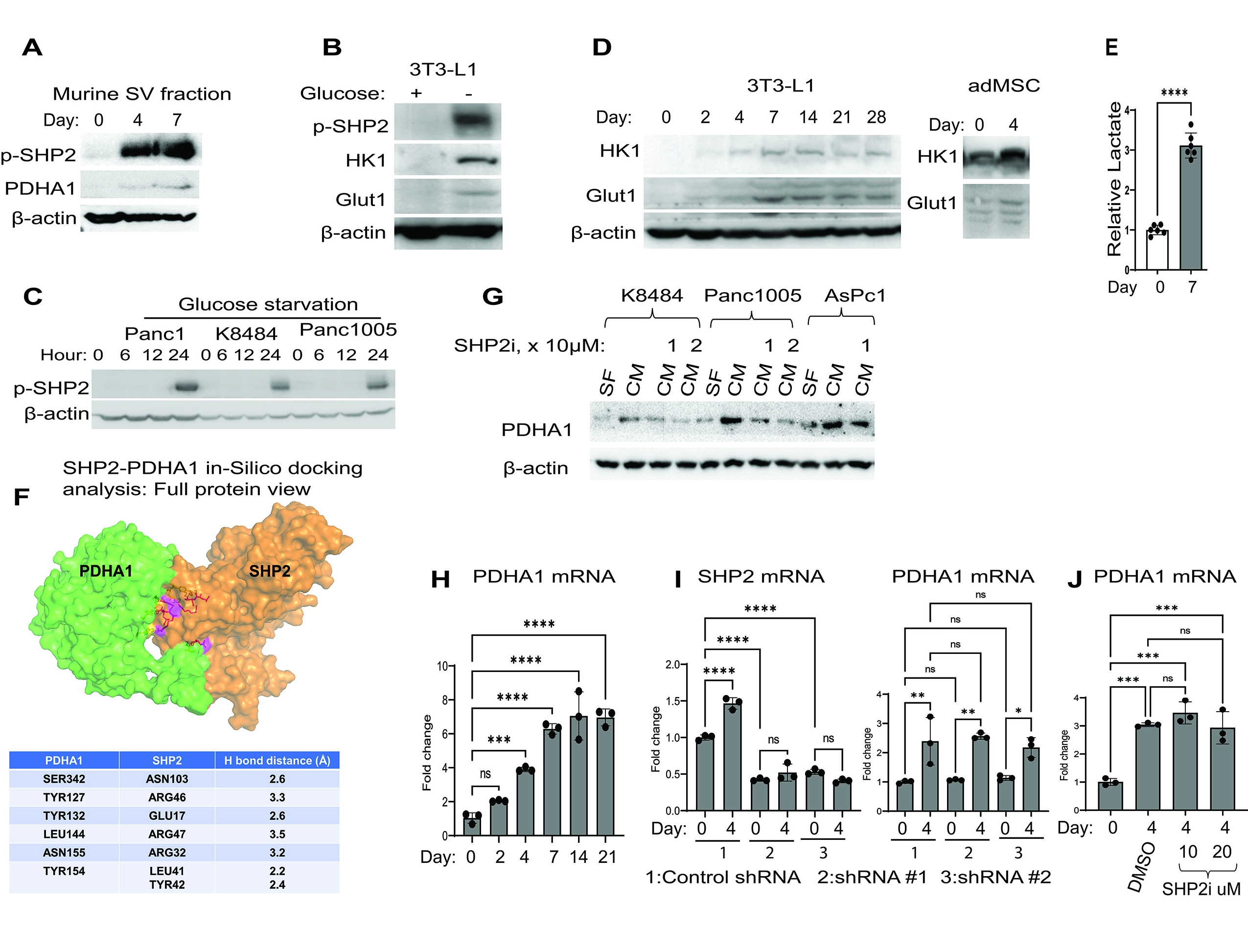

Supplement: Supplementary file 1 — Supplementary file1 (TIF 2177 kb) [file 12079_2022_691_MOESM1_ESM.tif]

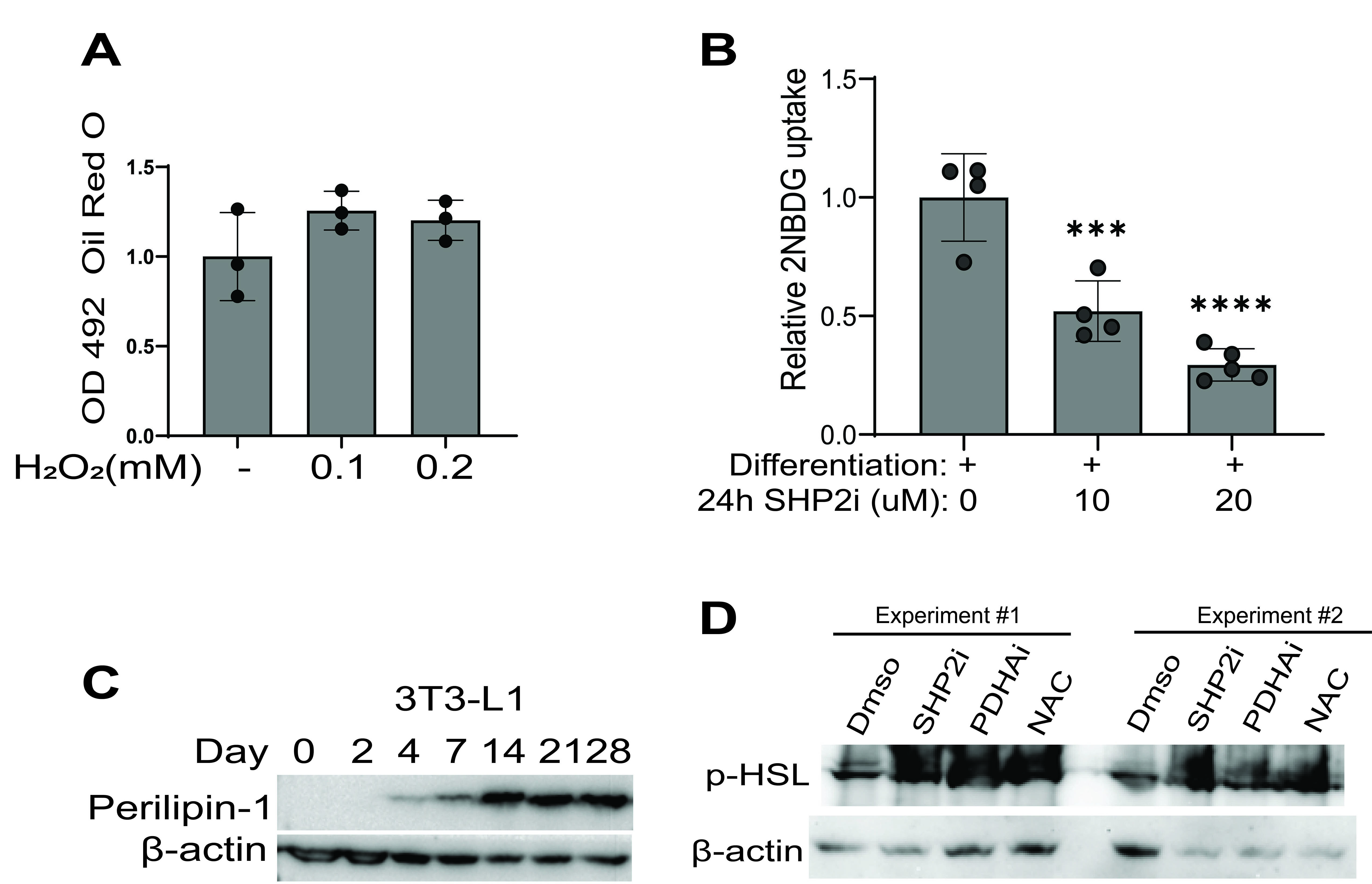

Supplement: Supplementary file 2 — Supplementary file2 (TIF 3427 kb) [file 12079_2022_691_MOESM2_ESM.tif]
