# Supplementary material for: SHP2 regulates adipose maintenance and adipocyte-pancreatic cancer cell crosstalk via PDHA1
Source: J Cell Commun Signal. 2022 Sep 8;17(3):575–90. doi: 10.1007/s12079-022-00691-1 (PMC10409927; doi:10.1007/s12079-022-00691-1)
Supplement: Supplementary file 3 — Supplementary file3 (TIF 36 kb) [file 12079_2022_691_MOESM3_ESM.docx]

**Supplementary Figure 1**: **A**: Mouse SV fraction cells were differentiated for the indicated time points followed by western blotting with indicated antibodies. **B-C**: 3T3-L1 (B) or indicated PDAC cells (C) were glucose-starved for 30h (B) or indicated time points (C) followed by western blotting with indicated antibodies. **D**: Indicated cells were stimulated with differentiation media as described in Figure 1C-D. Cell lysate was prepared at indicated time points followed by western blotting with indicated antibodies; the adMSC’s blots were run concurrently with the blots in Figure 1D and share the same actin control. **E**: 7-day differentiated 3T3-L1 cells were assessed for secreted lactate according to the manufacture manual (Cayman chemical, cat# 600450). **F:** Representation of in-silico analysis of SHP2:PDHA1 in surface view. The interaction of SHP2 with PDHA1 was studied using GrammX online server. The most favorable prediction was selected, and the interaction was studied using Pymol. The table identifies the interacting amino acids between SHP2 and PDHA1 with respective hydrogen bond distance. These hydrogen bond interactions (bond distance Å) include ASN103:SER342 (2.6 Å), ARG46:TYR127 (3.3 Å), GLU17:TYR132 (2.6 Å), ARG47:LEU144 (3.5Å), ARG32:ASN155 (3.2 Å), TYR42:TYR154 (2.4 Å) and LEU41:TYR154 (2.2 Å). Color scheme-Green: PDHA1 protein, Orange: SHP2 protein, Yellow: interacting amino acids of PDHA1, Pink: interfacing amino acids of SHP2. **G**: Conditioned media (CM) was collected from differentiated 3T3 (in complete media without serum) and applied to indicated cells (as described in the method section) for 48h followed by assessment of indicated proteins by western blot. Incubation with complete media without serum (SF) was used as negative control; treatment with SF or CM (alone) contained DMSO used as vehicle to dissolve SHP099; cells were pre-treated with SHP099 for 1h. **H**: Total mRNA was isolated at indicated time points, after induction of differentiation, followed by qPCR analysis with primers against indicated genes. **I**: SHP2 was knocked down in 3T3-L1 cells followed by differentiation for 4 days. Total mRNA was isolated and assessed by qPCR with primers against indicated genes. **J**: 3T3-L1 cells were stimulated in the presence of indicated concentrations of SHP2i for 4 days. Total mRNA was isolated at indicated time points followed by qPCR with primers against indicated genes. Error bars, SD; * P < 0.05.

**Supplementary Figure 2**: **A**: 3T3 cells were stimulated in the presence/absence of H2O2 for 5 days as described in Figure 3E&F; media replenished every 2 days. Lipid levels were quantified. **B**: 3T3 cells were differentiated for 5 days before being treated with SHP2i for 24h; 0 µM SHP2i contained DMSO as a control. The cells were then assayed for 2NBDG uptake as described in Figure 4A. **C**: Indicated proteins were assessed by western analysis at indicated time points after induction of differentiation in 3T3-L1 cells. **D**: Indicated proteins were assessed as described in Figure 5A.
